# Phytomedicine in Joint Disorders

**DOI:** 10.3390/nu9010070

**Published:** 2017-01-16

**Authors:** Dorin Dragos, Marilena Gilca, Laura Gaman, Adelina Vlad, Liviu Iosif, Irina Stoian, Olivera Lupescu

**Affiliations:** 1 Medical Semiology Department, Faculty of General Medicine, “Carol Davila” University of Medicine and Pharmacy, B-dul “Eroilor Sanitari” nr. 8, Sector 6, 76241 Bucharest, Romania; dordrag@drdorindragos.ro; 2 Nephrology Clinic, University Emergency Hospital Bucharest, 050098 Bucharest, Romania; 3 Biochemistry Department, Faculty of General Medicine, “Carol Davila” University of Medicine and Pharmacy, B-dul “Eroilor Sanitari” nr. 8, Sector 6, 76241 Bucharest, Romania; marilenagilca@gmail.com (M.G.); glauraelena@gmail.com (L.G.); 4 Physiology Department, Faculty of General Medicine, “Carol Davila” University of Medicine and Pharmacy, B-dul “Eroilor Sanitari” nr. 8, Sector 6, 76241 Bucharest, Romania; adelina_munteanu@yahoo.com; 5 R&D IRIST LABMED SRL, Str. Miraslau, nr. 24, Sector 3, 031235 Bucharest, Romania; iristlabmed@gmail.com; 6 Orthopaedic and Trauma Clinic 2, Faculty of General Medicine, “Carol Davila” University of Medicine and Pharmacy, 020022 Bucharest, Romania; olivera_lupescu@yahoo.com; 7 Clinical Emergency Hospital Bucharest, 014461 Bucharest, Romania

**Keywords:** osteoarthritis, rheumatoid arthritis, medicinal plants, herbs

## Abstract

Chronic joint inflammatory disorders such as osteoarthritis and rheumatoid arthritis have in common an upsurge of inflammation, and oxidative stress, resulting in progressive histological alterations and disabling symptoms. Currently used conventional medication (ranging from pain-killers to biological agents) is potent, but frequently associated with serious, even life-threatening side effects. Used for millennia in traditional herbalism, medicinal plants are a promising alternative, with lower rate of adverse events and efficiency frequently comparable with that of conventional drugs. Nevertheless, their mechanism of action is in many cases elusive and/or uncertain. Even though many of them have been proven effective in studies done in vitro or on animal models, there is a scarcity of human clinical evidence. The purpose of this review is to summarize the available scientific information on the following joint-friendly medicinal plants, which have been tested in human studies: *Arnica montana, Boswellia* spp., *Curcuma* spp., *Equisetum arvense, Harpagophytum procumbens*, *Salix* spp., *Sesamum indicum*, *Symphytum officinalis*, *Zingiber officinalis*, *Panax notoginseng*, and *Whitania somnifera*.

## 1. Introduction

Chronic joint inflammatory disorders such as osteoarthritis and rheumatoid arthritis have in common an upsurge of inflammation, and oxidative stress, resulting in progressive histological alterations and disabling symptoms.

Osteoarthritis, one of the most common musculoskeletal disorders, affecting approximately 15% of the population [1], is characterized by irreversible destruction of articular cartilage and bone erosion, induced by pro-inflammatory cytokines, e.g., interleukin 1 (IL-1), interleukin 6 (IL-6), and tumor necrosis factor α (TNF-α). These mediators increased the collagenase or matrix metaloproteinase (MMP) synthesis and the degradation of collagen type II, and decreased the synthesis of collagenase inhibitors, collagen and proteoglycans [2]. Degradation of collagen type II by collagenase-1 and collagenase-3 (also called MMP-13) represents one of the biochemical hallmarks of osteoarthritis [3].

Factors that increase the risk of OA are advanced age, sex, overweight, increased body mass index (BMI), genetics, ethnicity, diet, trauma, certain physical or occupational activities that imply biomechanical stress (e.g., pressure, load-bearing) across the joints [4,5,6]. Monitoring the OA evolution and therapy involves pain and physical function assessment for shorter studies, as well as joint imaging for longer studies (1 year or more). Pain is evaluated with visual analog scales (VAS), while the functional impairment with Western Ontario and McMaster Universities OA Index (WOMAC) [7]. Other useful assessment tools of functional impairment are Lequesne Functional Severity Index [8] and Karnofski Performance Scale Index [9].

Rheumatoid arthritis (RA) is a chronic progressive systemic autoimmune disease affecting 1% of the population and generating disability and increased risk for cardiovascular disease, lymphoma, and death [10], typically associated with high levels of oxidative stress and inflammatory mediators. RA is currently treated with a wide variety of medicines ranging from steroidal/nonsteroidal anti-inflammatory drugs (NSAID and pain killers), to potent biological agents targeting specific immune and inflammatory pathways, such as TNF-alpha (TNF-α) inhibitors and interleukin-1 receptor antagonists [11]. Among nonsteroidal anti-inflammatory drugs, acetaminophen is most frequently used in very high doses (4000 mg/day). Concerning the pain killers, tramadol is highly recommended, but also other opioids (e.g., morphine) [12]. Etanercept, infliximab and rituximab represent few examples of TNF-α inhibitors used for the treatment of severe RA [13,14]. Anakinra (an IL-1 receptor antagonist) [15] and methotrexat are other therapeutic choices for RA [16].

The biologic therapies have proven to be highly successful and effective in the majority of RA cases, including the severe ones.

Unfortunately, the use of standard drugs in arthropathies is accompanied by numerous and frequently serious side effects [17]: gastrointestinal ulcerations, hemorrhagic events, and nephrotoxicity induced by NSAID [18]; infusion hypersensitivity reactions, and auto-immune responses (e.g., lupus-like syndrome) triggered by TNFα inhibitors [19]; increased risk of severe infection, affecting mainly the respiratory tract, caused by biological drugs (anakinra, rituximab, or abatacept) [20]; fatal cytopenia induced by methotrexate [21]; etc.

Hence the renewed interest in medicines of botanical origin, which lack severe adverse effects and have a millennia-proven efficacy [22]. These remedies may be have a beneficial effect not only on the symptoms but also on the course of the disease [23].

The purpose of this review is to summarize the available scientific information obtained from medical databases and literature on medicinal plants that have been reported to have anti-arthritic activity in vitro, in animal models and also in human clinical studies.

A literature search was performed using the following phrases “medicinal plants or herb and osteoarthritis or arthritis or rheumatoid arthritis”, “*specific herb Latin name* or *specific herb English name* and osteoarthritis or arthritis or rheumatoid arthritis” (e.g., *Curcuma longa* or turmeric and osteoarthritis or arthritis or rheumatoid arthritis), in PubMed database. Only medicinal plants studied in human clinical studies were selected, and presented in alphabetical order of their Latin names. For all the plants included in the paper, we have analyzed in vitro studies, animal studies, and human clinical studies using herbal extracts, and potentially active phytochemicals. The corresponding papers were retrieved and evaluated in terms of the relevance for the present paper topic. Supplementary information was also obtained by a manual search in various books, including books of traditional medicine.

Several herbal extracts presented in the present paper (see Table 1) showed benefits in terms of pain and physical mobility, with low risk of side effects in arthritic subjects. These results warranting further investigation.

## 2. Anti-Arthritic Medicinal Plants

### 2.1. Arnica montana, family (fam.) Asteraceae

*Traditional knowledge*. This plant has been used for centuries in traditional herbalism as a remedy for trauma-, strain- and/or inflammation-related conditions of the locomotor system [24], and is one of the natural remedies most often used for rheumatologic conditions [25].

*Animal studies*. An orally administered *Arnica* extract was shown (on the collagen induced arthritis rat model) to alleviate both the histological and radiological changes in the affected joints, in parallel with a decrease in NO, TNF-α, IL-1β, IL-6, and IL-12 concentrations, anti-type II collagen antibodies level, and an improvement of the oxidative status (higher antioxidant levels and milder peroxidative injury) [24].

*Human clinical studies*. In an open multicenter trial, a gel prepared form *Arnica montana* fresh plant was tested in knee OA and proven to alleviate symptoms, improve functionality, and to be well tolerated. Rare adverse events were reported. Allergy might be a concern, as is fitting for a true Asteraceae herb [26]. A double-blind study on 204 patients comparing *Arnica montana* with ibuprofen in topical applications for hand OA found no difference in terms of efficiency and side effects (less frequent for *Arnica*) [27], a result corroborated by another study [28]. The equipotency of *Arnica* with NSAID in the local treatment of hand OA was acknowledged also by a Cochrane review [29].

*Active phytochemicals*. The anti-arthritic efficiency is attributed by some authors to a synergism of phenolic and flavonoid compounds, the dominant active principles, detected in a methanol extract, which was found efficient on a collagen-induced arthritis (CIA) rat model [24].

### 2.2. Boswellia spp., fam. Burseraceae

*Traditional knowledge*. Used for centuries in Ayurveda medicine (where it is called sallaki) *Boswellia serrata* (BS) yields a gum resin, known as frankincense, efficacious in the treatment of inflammatory disorders [30], particularly arthritis. Nowadays, many anti-arthritic combinations contain BS.

*In vitro studies*. A BS preparation enriched in active principles was able to hinder cartilage breakdown by metalloproteinase-3 (MMP-3) and to block Intercellular Adhesion Molecule 1 (ICAM-1) and thereby the inflammatory reaction [31]. In another study, a *B. frereana* preparation decreased the synthesis/ activation of several inflammation-related mediators and enzymes (MMP-9 and MMP-13, cycloxygenase-2, nitric oxide, prostaglandin E2), thus thwarting collagen and cartilage dissolution [32].

A poly-herbal formulation containing *Zingiber officinale* root, *Tinospora cordifolia* stem, *Phyllanthus emblica* fruit and oleoresin of BS has been shown to halt cartilage degradation in the knee (decreased release of glycosaminoglycans and aggrecan) associated with anti-inflammatory activity (as assessed by lower levels of nitric oxide) [33].

Another combination including three herbs (*Uncaria tomentosa*, *Boswellia* spp., and *Lepidium meyenii*) and an amino acid (l-leucine) has been shown to hamper inflammation and protect the articular cartilage. Tested on OA chondrocytes, it blocked the IL-1β-triggered activation of NF-κB and consequently abrogated the activity of inflammation-related enzymes (iNOS, MMP-9 and MMP-13), leading to a decreased rate of NO production and of cartilage matrix deterioration (less glycosaminoglycans-GAGs-released); simultaneously, enhanced production of structural proteins (including aggrecan and type II collagen) was detected [23].

*Animal studies*. Using the rat model of collagen induced arthritis, an extract of BS was able to suppress pro-inflammatory mediators and to improve the antioxidant status, as reflected by lactoperoxidase, myeloperoxidase, catalase, superoxide dismutase (SOD), glutathione (GSH), nitric oxide (NO) [22]. A mixture of *Withania somnifera*, BS, *Zingiber officinale* and *Curcuma longa* was tested on a rat model of adjuvant induced arthritis and proven to relieve inflammation and arthritis, and also to diminish the production of TNF-α and NO [34].

*Human clinical studies*. A Cochrane systematic review concluded that preparations from BS “show trends of benefits” (when used for the treatment of OA) coupled with a low burden of side effects, citing two high-quality and two moderate-quality studies demonstrating superiority compared to placebo in reducing pain and increasing functionality, and a moderate-quality study indicating a favorable adverse events profile [35]. Indeed, a couple of double-blind, randomized, placebo-controlled studies done on patients with knee OA demonstrated that phytopreparations from BS gum resin are able to reduce pain and increase functionality after only a few days (a week or so at most) with no serious adverse effects [31,36]. A phytosomal *Boswellia* preparation has been shown to be beneficial in the treatment of knee OA when added to the standard management of this condition (ameliorating the functional status (higher Karnofski Scale Index) and the symptoms (lower WOMAC (Western Ontario and McMaster Universities questionnaire) Score) [37]). It also hasten functional recovery and diminished pain and objective physical and humoral signs of inflammations in persons with arthritis of the hand induced by work-related overstraining [38]. A combination of *Curcuma longa* and BS was proven to be safe and efficient in patients with osteoarthritis, alleviating symptoms and objective signs, even better than celecoxib (a selective COX-2 inhibitor) and being practically devoid of side effects [39]. However other studies failed to confirm the efficiency of BS in the treatment of active RA [40].

*Active phytochemicals*. The role of bioactive principles is revendicated by the boswellic acids [41,42], a family of pentacyclic triterpenes, among which the main contenders were, initially, 11-keto-β-boswellic acid and acetyl-11-keto-β-boswellic acid. Oral or local administration of boswellic acids in a bovine serum albumin-induced arthritis model ameliorated the electrophoretic pattern of the synovial fluid proteins and decreased the infiltration of leucocytes into the knee joint [43]. These compounds were considered until recently to inhibit leukotriene synthesis by suppressing lipoxygenase-5 (LOX-5) in vitro and in animal models [30,44]. Other mechanisms were also invoked such as suppression of NF-κB activation, reduction of the production of pro-inflammatory cytokines (TNF-α, IL-1, IL-2, IL-4, IL-6 and IFN-γ), and reduced cleavage of C3 into the active components C3a and C3b, hampering the activation of the classic path of the complement [45]. These were showed in various in vivo experiments, with various arthritic or non-arthritic inflammation models (e.g., hypersensitivity reaction in mice) [46]. More recently, concerns were raised regarding both the action intensity of 11-keto-β-boswellic acid and acetyl-11-keto-β-boswellic acid, and their bioavailability, the latter being too poor to provide concentrations high enough for bioactivity [47]. Nonetheless, another member of the family was proposed as active principle, namely β-boswellic acid, able to reach much higher levels in plasma, enough to efficiently exert its inhibitory action on microsomal prostaglandin E synthase-1 and on the serine protease cathepsin G, which might be the substrate of the inflammation-suppressing aptitude of BS preparations [17].

### 2.3. Curcuma spp., fam. Zingiberaceae

*Traditional knowledge*. Several members of the *Curcuma* genus are used in traditional medicine, most important being *Curcuma longa* (CL), turmeric. Its rhizome has a centuries-long use as a dietary spice as well as an Ayurveda herb prized for its anti-inflammatory properties, hence its utility in arthritic conditions including RA [48].

*Animal studies*. In an animal arthritis model a preparation from CL lacking essential oil strongly suppressed joint inflammation and periarticular damage in correlation with decreased activation of NF-κB and of the ensuing cascade of events (involving mediators of inflammation and injury such as chemokines, cyclooxygenase 2, and receptor activator of nuclear factor kappa-B ligand (RANKL)) [49]. The ability to prevent the destructive changes in joints and periarticular bone seems to be comparable to that of betamethasone [50,51]. Liposomal encapsulation may help overcome the poor bioavailability problem generated by the low water-solubility [52]. The osteoclast-osteoblast balance is tipped in favor of bone building, while halting the OA progression [52]. In a rat-model of experimentally induced arthritis, a combination of ginger and turmeric rhizomes was superior to indomethacin (a potent NSAID) regarding the ability to alleviate both joint histopathological changes, and the extra-articular manifestations, including systemic inflammation (leukocytosis, thrombocytosis, and hyperglobulinemia), malnutrition (decreased body weight gain, and hypoalbuminemia), and iron deficiency anemia, with no prejudice to kidney function and reduced risk of cardiovascular disease (favorable lipid and oxidative profile) [53].

*Human clinical studies*. A mixture of *Curcuma longa* and *Boswellia serrata* has been shown to be more efficient than a standard dose of celecoxib (a selective COX-2 inhibitor) in the treatment of osteoarthritis, improving both objectively and subjectively the condition of the patients, with no toxicity detectable by clinical examination and laboratory tests (hemogram, liver and renal function tests) [39]. *Curcuma domestica* extracts have been shown to be useful in knee osteoarthritis, reducing the pain and preserving functionality with an efficiency equivalent to ibuprofen, but with fewer gastrointestinal side effects [54]. A recent meta-analysis found relevant scientific evidence for the efficacy of turmeric as a therapeutic option in arthritis, but concluded that more studies are necessary in order to definitively pin it down [55].

*Active phytochemicals*. The active ingredient is diferuloylmethane, a yellow phenolic pigment commonly known as curcumin, which has a multifaceted beneficial action in various fields of pathology (diabetes, cancer, inflammation, oxidative stress) due to its ability to favorably influence a variety of signaling pathways and mediators [56]. In a rat model of arthritis, it has been shown to ameliorate the joint inflammation (evaluated by the neutrophil infiltrate density), in the first six hours after the arthritis-inducing event (zymosan infiltration) being even more effective than a low dose of prednisone [57].

The reduction of the systemic oxidative stress as reflected by enhanced serum SOD activity, increased GSH level, and decreased malondialdehyde level was considered part of the mechanism of action [58]. The blockage of glial activation resulting in decreased synthesis and secretion of inflammatory mediators in the spinal cord, corroborated by similar results in studies on cultures of astrocytes and microglia can be responsible for arthritic pain reduction [59].

β-Elemene, found in *Curcuma Wenyujin*, an herb used in Traditional Chinese Medicine for the treatment of rheumatoid arthritis, may be another phytochemical active in arthropathies. This compound antiproliferative activity (useful in neoplastic diseases) may explain the suppressive effect on accumulation of fibroblast-like synoviocytes. One in vitro study showed that the underlying mechanism may be represented by the apoptosis induction via increased production of reactive oxygen species and activation of p38 mitogen-activated protein kinase (MAPK) [60].

### 2.4. Equisetum arvense, fam. Equisetaceae

*Traditional knowledge*. *Equisetum arvense*, also known as horsetail, has a long history of use in European ethnomedicine as an anti-inflammatory remedy [61].

*Animal studies*. An animal model of arthritis induced through an antigen challenge was used to prove the downregulating effects of *Equisetum giganteum* on lymphocyte proliferation. B and T lymphocytes are both affected by the immunomodulatory action of this plant, which is nevertheless free of cytotoxicity [62].

*Human clinical studies*. The beneficial effect of horsetail in RA was substantiated by a study that pointed out the decrease in TNF-α as one of the contributing mechanisms [63].

*Active phytochemicals*. Kynurenic acid was proposed as a putative mediator of the anti-inflammatory and antalgic effect of several herbs beneficial in RA, horsetail among them [64]. The anti-inflammatory potential of kynurenic acid was showed until now only in non-arthritic animal models (e.g., acute experimental colitis in rat) [65]. Kynurenic acid is also an endogenous oxidative metabolite of tryptophan, with glutamate-receptor antagonist activity, which may explain partially its analgesic properties. One in vitro study showed that kynurenic acid is able to inhibit the proliferation of synoviocytes [66]. Its level was significantly lower in human subjects with RA than that in those with OA [67], fact which suggests the beneficial potential of kynurenic acid supplementation in RA patients.

### 2.5. Harpagophytum procumbens, fam. Pedaliaceae

*Traditional knowledge*. *Harpagophytum procumbens* (HP), also known as devil's claw, a medicinal plant native to Africa, is a “celebrity” among anti-osteoarthritis natural remedies, being approved by German Commission E for the treatment of degenerative diseases of the musculoskeletal system [68].

*In vitro studies*. HP extracts showed chondroprotective activity, several mechanisms being potentially responsible for this: decreased synthesis of inflammatory mediators (e.g., TNF-α, and interleukin-1β), and inhibition of matrix metalloproteinases and elastase [69].

*Animal studies*. A dried aqueous extract of HP has been shown to have a significant dose-dependent analgesic and anti-inflammatory activity in rats at 5 and 10 mg/kg. Nevertheless, carrageenan-induced paw edema was not affected by the isolated harpagoside constituent. This signifies that harpagoside may not have an anti-inflammatory effect, at least in the dosage used in this study [70,71]. This suggests that other HP constituents may be responsible for the anti-inflammatory effect.

*Human clinical studies*. Several human clinical studies showed that various HP tuber extracts (equivalent to 50–60 mg harpagoside daily, administered for a variable period between 8 and 16 weeks, depending on the study) significantly improved the clinical picture of subjects with knee and hip osteoarthritis in terms of pain, movement limitation, and joint crepitus [72,73,74]. The severity of pain and other symptoms was assessed by the WOMAC questionnaire, VAS, Lequesne Index, physician exam and/or pain-relieving medication dose.

*Active phytochemicals*. The major phytochemicals responsible for the anti-osteoarthritis effect are the iridoid glycosides (harpagoside, harpagide, and procumbide), which are found in a higher amount in tubers and root. Although, it is worthy to mention that the whole-plant extracts seem to have a better therapeutic effect than those obtained from isolated parts [75]. Harpagoside inhibited in vitro the synthesis of various pro-inflammatory mediators via suppression of iNOS and COX-2 expression through inhibition of NF-κB activation [76], but showed no anti-inflammatory activity in one animal model of inflammation (carrageenan-induced paw edema) [70]. The scientists suggested that other phytochemicals than harpagoside may be contributors to the anti-inflammatory effect of HP [70,77].

### 2.6. Panax notoginseng, fam. Araliaceae

*Traditional knowledge*. *Panax notoginseng* (PN), known as sanqi in Chinese, has a long history of clinical use for the treatment of traumatic injuries, swellings and pains [78].

*In vitro studies*. An n-butanol extract of PN inhibited the synthesis of pro-inflammatory mediators (TNF-alpha, IL-1, inducible NOS, and MMP-13) in vitro [79].

*Animal studies.* PN in combination with other two herbs (*Rehmannia glutinosa* and *Eleutherococcus senticosus*), had a suppressive effect on collagen-induced arthritis in mice, through inhibition of TNF-alpha, IL-1, iNOS, and MMP-13 synthesis [80].

*Human clinical studies*. The same combination of PN with *Rehmannia glutinosa* and *Eleutherococcus senticosus*, administered as capsules, 4 capsules of 400 mg daily for six weeks (containing ginsenoside Rb1, 19.49 ± 3.89 mg/g, stachyose 0.87 ± 0.17 mg/g and eleutheroside E 0.07 ± 0.014 mg/g) improved the physical function and pain according to the Korean version of WOMAC questionnaire in 57 patients with knee OA, and was considered effective for symptomatic relief [2].

*Active phytochemicals*. Saponins are considered the main osteo-active phytochemicals from PN. PN saponins promoted the autograft tendon healing in bone tunnel [81], and amplified the effect of routine therapy (diclofenac sodium, Leflunomide and prednisone) in terms of joint swelling, tenderness, and pain index, as well as time of morning stiffness, VAS and immunological parameters, in patients with RA [82].

### 2.7. Salix spp., fam. Salicaceae

*Traditional knowledge*. Various species from the genus *Salix*, or willow, were already in common use for pain relief in antiquity [83,84], being first mentioned in the Ebers papyrus (about 1550 BC) [85], and afterwards by all the great masters of medicine in antiquity, middle ages, and modern times [86].

*In vitro studies*. The inflammation-suppressing effect of willow bark extract (WBE) relies, at least partially, on its ability to antagonize the activated monocytes, by blocking the activity of pro-inflammatory cytokines (TNFα), enzymes (COX-2), and mediators (NF-κB) [87].

*Animal studies*. The mechanism of the anti-inflammatory action of WBE was examined on two animal models of arthritis, an acute and a chronic one. WBE decreased the inflammatory infiltrate and exudate and blocked the cytokine surge with a potency at least equivalent to that of acetylsalicylic acid (ASA), was better than ASA in reducing leukotrienes levels and in inhibiting COX-2, and as good as ASA in decreasing prostaglandins levels. WBE influenced favorably the oxidative stress increasing GSH and decreasing malondialdehyde levels more efficiently than ASA or celecoxib (a selective COX-2 inhibitor). Despite being more potent than ASA, on a molar basis, the salicin amount in WBE is much less than the salicylate content of ASA, suggesting that active principles other than salicin might play a role in the anti-inflammatory and antioxidative action of WBE, the polyphenols being among the candidates, at least regarding the protection against free radicals [88]. The ability to mitigate pro-inflammatory cytokines and oxidative stress was corroborated by still another study on the collagen induced arthritis animal model [89].

*Human clinical studies*. The first clinical trial of aspirin, albeit uncontrolled and non-randomized, was conducted in the 18th century by the English Reverend Edward Stone—the good fellow, struck by the quinine-like bitterness of aspirin, surmised an antifebrile activity and, indeed, was able to cure fever in 50 patients [86]. A two-week, double-blind, randomized, placebo-controlled trial demonstrated the ability of willow bark extract (in a dose equivalent to 240 mg salicin/day) to control the symptoms of patients with OA, especially to reduce pain, although with rather subdued efficiency [90]. The same dose of willow bark extract was used in two other six-week, randomized, controlled, double-blind trials in patients with OA and RA, respectively, the herbal preparation being compared with a potent NSAID (diclofenac) and with placebo. The two trials yielded sobering results, as in neither was willow bark extract significantly better than placebo in pain relief [91]. In a six-week, open, multicentric observational study with reference treatment, WBE was evaluated as better as conventional therapy by physicians and patients alike, in terms of both therapeutic efficiency and side effects, when used for hip and knee degenerative disease [92].

A systematic review concluded that there is moderate evidence for the efficiency of WBE in low back pain, but insufficient data for OA and RA, suggesting that higher doses should be tested [84].

In a longer (six months) observational study on 436 patients with OA and back pain, WBE significantly decreased pain and was well tolerated [93].

*Active phytochemicals*. Although traditionally salicin was considered as the active principle, there are opinions that this substance cannot explain the whole range of effects of WBE, and that other phytochemicals might be involved, such as polyphenols and flavonoids, which showed inhibitory activity on COX-2 and decreased synthesis of pro-inflammatory mediators in vitro, in human monocytes and differentiated macrophages [87,88,94,95,96].

### 2.8. Sesamum indicum, fam. Pedaliaceae

*Traditional knowledge*. Sesame oil (SO) extracted from *Sesamum indicum* (SI) has been used in various Asian traditional medicines to alleviate pain in inflammatory conditions of the joints, teeth, skin, etc. [97].

*Animal studies*. SO was tested in a rat model of adjuvant (Freund’s adjuvant)-induced arthritis and was able to dampen the biochemical consequences of oxidative stress: lower plasmatic levels of thiobarbituric acid reactive substances and reduced gamma-glutamyltransferase activity in the joints and spleen [98]. In a rat model of acute gout-like arthritis, SO strongly hindered the inflammatory reaction, thinning the inflammatory infiltrate, lowering the levels of inflammatory mediators (TNF-α, IL-1β, IL-6), impeding the activity of nuclear factor-κB (NF-κB) (at least in the mast cells) and complement system activation [97]. In another rat model of OA, SO alleviated joint pain by inhibiting oxidative aggression (decline in the peroxidation of lipids and in the production of superoxide anion and peroxynitrite, and a surge in glutathione and glutathione peroxidase levels) in the muscles associated with nuclear factor erythroid-2-related factor [1]. SO is active in experimentally induced arthritis through its minor constituents (devoid of these minor constituents it is inactive), decreasing not only the clinically visible joint inflammation, but also its serum markers (oxidative stress related molecules, RA markers, inflammatory eicosanoids and cytokines) and the activity of hydrolytic enzymes; additionally, bone loss was also diminished [99].

*Human clinical studies*. In a study on patients with knee OA, oral administration of sesame together with standard therapy produced better outcomes in terms of objective and subjective manifestations compared to standard therapy alone [100]. In a placebo controlled trial on patients with knee OA, sesame seed administration was associated with a statistically significant drop in serum levels of malondialdehyde and of high-sensitivity C-reactive protein (hs-CRP) after two months of treatment. and significantly lowered levels of IL-6 after treatment [101].

*Active phytochemicals*. The ability of SI to protect from the dire consequences of inflammation and oxidative stress (aging, cancer, cardiovascular disease, to name only a few) seems to be due to the lignans. It contains sesamin and its hydroxylated counterpart, sesamolin. Similar biological activity has a phenolic compound, sesamol (3,4-methylene-dioxy-phenol) which results from the degradation of sesamolin [102]. Sesamol has been proven to mitigate joint inflammation, cartilage degradation, and periarticular bone resorbtion in adjuvant-induced arthritis animal model. This action was paralleled by a drop in the level of pro-inflammatory cytokines and in the activity of tissue-destructive enzymes [103]. In addition, a restoration of the oxidant homeostasis reflected in decreased oxidative stress markers and a boost in the activity of protective enzymes were noticed [103]. The hydroperoxides-scavenging capacity of sesamol makes it able to arrest the oxidation state of iron and consequently the conversion of inactive LOX (Fe^2+^) to active LOX (Fe^3+^), which leads to the inhibition of this inflammation-promoting enzyme [102].

In a study done on porcine cartilage explant exposed to the pro-inflammatory action of TNF-α and oncostatin M (as an animal RA model), sesamin has been proven to preclude the cytokine-induced cartilage degeneration by slowing down the degradation of constitutive glycosaminoglycans and collagen [104].

### 2.9. Symphytum officinalis, fam. Boraginaceae

*Traditional knowledge*. *Symphytum officinalis*, also known as comfrey, is a medicinal plant traditionally used in Europe for the treatment of inflammatory disorders [105,106].

*In vitro studies*. An extract of comfrey significantly inhibited the respiratory burst of polymorphonuclear leukocytes, suggesting an anti-inflammatory potential [107].

*Animal studies*. Comfrey extracts showed anti-inflammatory activity, by *inhibit*ing *carrageenan-induced rat paw oedema* [108,109].

*Human clinical studies*. A study on people aged 50–80 with OA of the knee proved that topically applied comfrey preparation decreased pain, although was unable to decrease the burden of inflammatory molecules or the rate of cartilage breakdown, the only noticeable adverse effect being local rash [110].

Similar results yielded another study on a similar population of years-long sufferers from OA of the knee: a comfrey-containing ointment improved the quality of life by decreasing pain and increasing knee-mobility [111].

*Active phytochemicals*. Phenolic acids (e.g., rosmarinic acid), glycopeptides and amino acids are considered to be, at least in part, responsible for the anti-inflammatory potential of comfrey root extracts, in various vitro models [108,112]. Rosmarinic acid inhibited prostaglandin synthesis, and carrageenan- and gelatine-induced erythrocyte aggregation [113].

### 2.10. Zingiber officinalis, fam. Zingiberaceae

*Traditional knowledge*. *Zingiber officinalis* (ZO), also known as ginger, is a common spice used in Asian cuisine, and a traditional remedy for joint diseases in ethnomedicine [48].

*In vitro studies*. ZO is thought to have anti-inflammatory effects, possibly by inhibiting COX-1, COX-2 and LOX [114,115,116]. Nevertheless, the squeezed ginger extract paradoxically increased the synthesis of pro-inflammatory cytokines (TNF-α, IL-6, and monocyte chemotactic protein-1) in RAW 264 cell culture [117].

*Animal studies*. The oral administration of the squeezed ginger extract had a dual effect on TNF-α synthesis in mice, in peritoneal cells: ZO extract initially augmented it, but after repeated administrations decreased it [117]. In addition, it augmented the serum corticosterone level, and this may contribute to the anti-inflammatory effect of ZO.

*Human clinical studies*. A recent study found that ZO powder supplementation (1 g/day) for three months can reduce the serum level of nitric oxide and high-sensitivity reactive protein hs-CRP in patients with knee OA. The inflammatory markers started to decrease after three weeks of treatment [118]. Several other studies showed clinical improvement in OA patients with ZO extract, as evaluated by the pain score with VAS, reduction in intake of rescue medication, having mostly mild gastrointestinal adverse events, and similar or even better efficacy and satisfaction score than the standard treatment prescribed by the orthopedic specialist [119,120,121].

Another study found that the oral daily administration of one ZO preparation (340 mg EV.EXT 35 *Zingiber officinalis* extract) and glucosamine (1000 mg) for four weeks, in 21 patients with confirmed knee and hip OA, significantly reduced the arthritic pain on standing and moving, according to VAS scale evaluation. Moreover, this treatment had a similar efficacy as diclofenac (100 mg/day) plus glucosamine (1000 mg/day), but a higher safety, due to the decrease of gastrointestinal pain and increase of gastroprotective prostaglandin (PGE1, PGE2, and PGF2α) levels in the stomach mucosa [122]. However, one cross-over study (a wash-out period of one week followed by three treatment periods of three weeks duration each) found no significant difference between placebo and ginger extract in OA patients [123].

*Active phytochemicals*. Pungent constituents of ZO were thought to contribute to the anti-inflammatory activity of this medicinal plant. For instance, 1-dehydro-[10]-gingerdione inhibited κB kinase β activity required for NF-κB activation and suppressed NF-κB-regulated expression of inflammatory genes in lipopolysaccharide S-activated macrophage [124]. 6-Dehydrogingerdione attenuated iNOS, COX-2, IL-1β, IL-6, and TNF-α gene expression in vitro, in RAW 264.7 macrophages. [114]. Other compounds from ZO (10-gingerol, 8-shogaol and 10-shogaol) showed the capacity to decrease COX-2 activity in vitro [115].

### 2.11. Whitania somnifera, fam. Solanaceae

*Withania somnifera* (WS), also known as ashwagandha, is a potent anti-osteoarthritic and anti-inflammatory plant used in Ayurveda [125].

*In vitro.* One study showed that the WS extract inhibited liposaccharyde S induced synthesis of pro-inflammatory cytokines (TNF-alpha, IL-1beta and IL-12) in peripheral and synovial fluid mononuclear cells from rheumatoid arthritis subjects in vitro, but had no effect on IL-6 synthesis [126]. The WS extract also showed inhibitory effects on collagenase activity against the degradation of the bovine Achilles tendon type I collagen, that may be useful in joint disease treatment [127].

*Animal studies*. WS root powder had protective effect on bone collagen in experimental induced arthritis model in rats [128].

*Human clinical studies*. A randomized, double blind placebo controlled study showed that the aqueous extract of WS produced significant reduction of scores for pain, stiffness and disability in human subjects with knee joint pain [129].

*Active phytochemicals*. Withaferin A, belonging to the steroid class of phytochemicals, is thought to be one of contributor compounds to the beneficial effects of WS in OA subjects [126]. Whitaferin A reverted to near normal levels the increase in paw volume, lysosomal enzymes, lipid peroxidation, and TNFα in an monosodium urate crystal-induced arthritis in mice [130]. Withaferin A supressess NF-κB activation by targeting a crucial cysteine 179 in IκB kinase β, and by inhibition of the NF-κB Essential Modulator/ IκB kinase β association complex formation, according to molecular docking and molecular dynamics simulations studies [131,132].

## 3. Concluding Remarks

Several medicinal plant extracts showed trends of clinical and biochemical benefits with low risk of side effects in arthritic patients that warrant further investigation, including imagistic and histological evaluation. The search for effective herbal supplements as a complementary therapy of degenerative arthropathies is a complex issue and implies a long process, in the end of which the conclusions are difficult to be drawn, due to the differences in terms of study design and protocols [133]. The available studies did not evaluate the effect of plant extracts on disease progression, or whether it halts the aggravation of arthropathies. For this purpose, studies of longer duration should be performed.

Almost no studies confirmed in humans the biological mechanisms of herbal extracts found in vitro or in animal studies, the majority of the available ones being focused only on the evaluation of the symptomatic relief induced by the herbal treatment.

Despite their advantages, herbal treatments raise several concerns, such as drug–herbal interactions, low bioavailability, lack of standardization, insufficient regulatory guidelines at national and international levels, and therefore possibility of adulteration [134,135,136,137].

More evidence of medicinal plant efficacy, safety and mechanisms of action are needed before herbal treatment can gain a place in therapeutic guidelines of OA and RA.

## Figures and Tables

**Table 1:** Medicinal plants with therapeutic potential in ostheoarthritis and rheumatoid arthritis (Legend: AM, animal model; CAT, catalase; COX, cyclooxygenase; GPx, glutathione peroxidase; GSH, glutathione; GST, glutathione-S-transferase; HS, human study; IL, interleukine; iNOS, inducible nitric oxide synthase; LOX, lipooxygenase; PGE1-S, prostaglandin E2 synthase; ROS, reactive oxygen species; SOD, superoxide dismutase; MAPK, mitogen-activated protein kinase; MCP-1, monocyte chemoattractant protein-1; MIP-1α, monocyte inflammatory protein-1; MMP, matrix metaloproteinase; NO, nitric oxide; TNF, tumoral necrosis factor; (−), decreased synthesis/decreased activation/inhibition of various mediators, enzymes, transcription factors, and processes; (+), increased synthesis/increased activation of various mediators, enzymes, transcription factors, and processes). Note: References in the table correspond only to the mechanism of action.

Plant	Active Phytochemicals	Mechanism of Action	References
*Arnica montana*	phenols, flavonoids	(−) NO, TNF-α, IL-1β, IL-6, IL-12, anti-type II collagen antibodies, (+) antioxidants (AM)	[24]
*Boswelia* spp.	boswelic acids	(−) PGE1-S, cathepsin G, LOX-5, MMP-9, MMP-13, COX-2, NO, PGE1, TNF-α, IL-1, IL-2, IL-4, IL-6, IFN-γ (in vitro, AM)	[17,30,31,41]
		(−) leukocyte infiltration in knee (AM)	[43]
*Curcuma* spp.	curcuminoids	(+) SOD, GSH, (−) MDA (HS)	[58]
		(−) neutrophil infiltrate in knee, (AM), (−) IL-1β, TNFα, MCP-1, and MIP-1α (in vitro, AM)	[57,59]
	β-elemene	(+) p38 MAPK (in vitro)	[60]
*Equisetum arvense*	kynurenic acid	(−) synoviocyte proliferation (in vitro)	[64,66]
*Harpagophytum procumbens*	iridoid glycosides	(−) iNOS and COX-2 (in vitro)	[76]
*Panax notoginseng*	saponins	(−) TNF-alpha, IL-1, iNOS, MMP-13 (AM)	[79,80]
*Salix* spp.	salicin, polyphenols, flavonoids	(−) TNFα, COX-2, IL-1, IL-6 (in vitro)	[87,95]
*Sesamum indicum*	sesamin, sesamol, sesamolin	(−) thiobarbituric acid reactive substances, LOX (in vitro), TNF-α, IL-1β, IL-6, hyaluronidase, MMP-13, MMP-3, MMP-9, exoglycosidases, cathepsin D, phosphatases, COX-2, PGE2, ROS, H2O2, MDA (AM), IL-6 (HS)	[1,97,98,101,102,103]
		(+) GSH, GPx (AM)	
*Symphitum officinalis*	rosmarinic acids, glycopeptides, amino acids	(−) PG (in vitro)	[108,112,113]
*Zingiber officinalis*	gingerdione derivatives, 10-gingerol, 8,10-shogaol	(−) COX-1, COX-2, LOX, iNOS, TNF-α, IL-1β, IL-6, MCP-1, κB kinase β (in vitro, AM), NO (HS)	[114,115,117,118,124]
		(+) cortisone (AM)	
*Whitania somnifera*	whitaferin A	(−)TNF-alpha, IL-1β, IL-12, collagenase (in vitro), NF kB (docking studies)	[126,127,131,132]
